# The Relationship between Type D Personality, Affective Symptoms and Hemoglobin Levels in Chronic Heart Failure

**DOI:** 10.1371/journal.pone.0058370

**Published:** 2013-03-05

**Authors:** Nina Kupper, Aline J. Pelle, Balázs M. Szabó, Johan Denollet

**Affiliations:** Center of Research on Psychology in Somatic diseases (CoRPS), Tilburg University, Tilburg, The Netherlands; 2 Department of Cardiology, TweeSteden Hospital, Tilburg, The Netherlands; 3 Department of Cardiology, St. Elisabeth Hospital, Tilburg, The Netherlands; Max Planck Institute of Psychiatry, Germany

## Abstract

**Background:**

Anemia is associated with poor prognosis in heart failure (HF) patients. Contributors to the risk of anemia in HF include hemodilution, renal dysfunction and inflammation. Hemoglobin levels may also be negatively affected by alterations in stress regulatory systems. Therefore, psychological distress characterized by such alterations may adversely affect hemoglobin in HF. The association between hemoglobin and Type D personality and affective symptomatology in the context of HF is poorly understood.

**Aim:**

To examine the relationship between Type D personality and affective symptomatology with hemoglobin levels at inclusion and 12-month follow-up, controlling for relevant clinical factors.

**Methods:**

Plasma levels of hemoglobin and creatinine were assessed in 264 HF patients at inclusion and at 12-month follow-up. Type D personality and affective symptomatology were assessed at inclusion.

**Results:**

At inclusion, hemoglobin levels were similar for Type D and non-Type D HF patients (*p* = .23), and were moderately associated with affective symptomatology (*r* = –.14, p = .02). Multivariable regression showed that Type D personality (*β* = –.15; *p* = .02), was independently associated with future hemoglobin levels, while controlling for renal dysfunction, gender, NYHA class, time since diagnosis, BMI, the use of angiotensin-related medication, and levels of affective symptomatology. Change in renal function was associated with Type D personality (*β* = .20) and hemoglobin at 12 months (*β* = –.25). Sobel mediation analysis showed significant partial mediation of the Type D – hemoglobin association by renal function deterioration (*p* = .01). Anemia prevalence increased over time, especially in Type D patients. Female gender, poorer baseline renal function, deterioration of renal function and a longer HF history predicted the observed increase in anemia prevalence over time, while higher baseline hemoglobin was protective.

**Conclusion:**

Type D personality, but not affective symptomatology, was associated with reduced future hemoglobin levels, independent of clinical factors. The relation between Type D personality and future hemoglobin levels was mediated by renal function deterioration.

## Introduction

Anemia is a common comorbidity in chronic heart failure (HF). In patients with comorbid kidney disease, more severe HF symptoms and in older patients, the prevalence of anemia ranges from 30 to 61%. In ambulatory HF patients with less severe HF symptoms (e.g. NYHA class I & II) the prevalence of anemia ranges from 4 to 23% [1]. Anemia is associated with symptoms of HF, such as dizziness, tachycardia, and dyspnea [2], as well as more frequent hospitalization [3], reduced health-related quality of life [4], and increased risk of mortality [5], [6]. The prevalence of anemia is closely related to the level of New York Heart Association (NYHA) functional class involved [7], indicating that anemia becomes more prevalent when HF becomes more severe and more symptomatic. The incidence and severity of anemia has also been associated with the progression of chronic renal dysfunction, another common comorbidity in HF [8].

In the majority of cases, anemia develops in HF patients as a result of their chronic disease [1]. Anemia in HF may have multiple origins, which are thought to involve reduced erythrocyte production, decreased body mass index (BMI) and hemodilution [1]. Further contributors to the risk of anemia in HF are comorbid renal disease and increased inflammation. Renal dysfunction may lead to a decrease in erythropoietin levels, and a subsequent decrease in bone marrow erythrocyte production [9]. Elevated levels of pro-inflammatory cytokines may also inhibit hematopoietic proliferation [10] which, in turn, causes anemia [9], also in patients with HF [11]. Another potent factor in the development of anemia (or pseudo-anemia) is hemodilution, due to increased plasma volume [12]. Finally, medication affecting the renin-angiotensin system (i.e. ACE inhibitors and angiotensin receptor blockers) reduces erythropoietin production and lowers hemoglobin levels [1].

In addition to these physiological mechanisms, animal research shows that psychological stress may also promote anemia. In rodents, acute psychological stress induced a decrease in blood and bone marrow iron and inhibited erythropoiesis [13], [14], while chronic psychological stress was associated with even lower plasma iron levels [14]. In humans, there is also a link between anemia and psychological factors. Even though no study so far has examined the effects of (chronic) stress on hemoglobin levels in human populations, other psychological factors such as depressed mood and diminished quality of life were associated with anemia and decreased hemoglobin level in COPD patients [15] and in community-dwelling elderly populations [16], [17]. Decreased hemoglobin levels and increased anemia were also observed in cancer patients who have difficulties in understanding and expressing their emotions (alexithymia) [18]. Conversely, treatment with erythropoietin analogues may improve quality of life and reduce depressive symptoms in anemic HF [19] and cancer [20], [21] patients.

Several forms of emotional distress such as depression, anxiety, and distressed or Type D personality, have been related to the above-mentioned pathophysiological mechanisms of anemia. Both Type D personality (i.e. the combination of negative affectivity and social inhibition [22]) and depression have been related to heightened pro-inflammatory activity [23], [24] in patients with chronic HF. Inflammation is known to adversely affect the hematopoietic system and hemoglobin balance [9]. Deregulation of HPA axis activity is another mechanism by which hemoglobin levels could be affected. Chronic cortisol exposure may suppress erythropoiesis [25], potentially by the down-regulation of erythropoietin mRNA expression in the kidneys [26], and may promote iron deficiency [27], thereby increasing the risk of anemia. Both depression [28] and Type D personality [29], [30] have been associated with hyperactivity of the HPA axis, resulting in a higher cortisol output. These studies showed that Type D personality was independently associated with a larger cortisol awakening response in patients hospitalized for acute coronary syndrome (ACS) [30] and with a larger daytime cortisol output in ACS outpatients, four months after their hospitalization for ACS [29], while adjusting for important covariates and depression levels. Moreover, in HF patients, larger cortisol/DHEA ratios have been related to lower hemoglobin levels [31], suggesting that glucocorticoid imbalance may also be involved in stress-related anemia in HF patients.

To date, the pathophysiological mechanisms through which emotional distress could affect prognosis in patients with HF are largely unknown. Since anemia negatively affects HF prognosis, the current study prospectively examined the association of Type D personality and affective symptoms with hemoglobin levels and renal function in HF patients.

## Methods

### Ethics Statement

The study protocol was approved by the Medical Ethics Committees of both hospitals and was conducted in accordance with the most recent Helsinki Declaration (2008). All patients provided written informed consent.

### Participants and Procedure

The total sample comprised 313 consecutive HF outpatients (response rate = 77%) recruited between October 2003 and June 2007 from the St. Elisabeth Hospital and the TweeSteden Hospital, both of which are teaching hospitals in Tilburg, the Netherlands.

Inclusion criteria consisted of left ventricular ejection fraction (LVEF) ≤40%; age ≤80; NYHA class I-III; no hospital admissions in the month prior to inclusion, and being stable on oral medication for one month prior to inclusion. Patients were excluded if they exhibited other life-threatening comorbidities (e.g., cancer); evident cognitive impairments; psychiatric comorbidity (except for mood disorders); a poor command of the Dutch language, or signs of acute infection at the time of blood sampling. The mean age of the total sample was 65.9±9.9 years, and 74.8% of these patients were male.

With regard to participation, patients were approached by their attending cardiologist or specialized heart failure nurse. At inclusion, patients were asked to fill out a questionnaire at home to assess socio-demographic variables, Type D personality and affective symptomatology. Questionnaires were returned in a stamped, self-addressed envelope. All questionnaires were checked for completeness. Any participants who had submitted incomplete questionnaires were contacted by phone or mail, and asked for details of the missing items. Any patients who failed to return the questionnaire within two weeks received a reminder phone call or a letter. Blood samples were collected to determine creatinine and hemoglobin levels at inclusion and at 12-month follow-up.

### Measures

#### Socio-demographics and clinical variables

Gender, age, educational level, and marital status were assessed by means of specially-designed questions in the survey. Clinical variables were obtained from the patients’ medical records. These comprised smoking status, body mass index (BMI), LVEF, NYHA, HF etiology, time since diagnosis, cardiac history (previous myocardial infarction, coronary artery bypass grafting or percutaneous coronary intervention), device therapy (implantation of an implantable cardioverter defibrillator, biventricular pacemaker or pacemaker), and comorbidities (history of stroke or transient ischemic attack, chronic obstructive pulmonary disease (COPD), diabetes, hypercholesterolemia, hypertension, peripheral arterial disease, and gastro-intestinal diseases). Finally, information on prescribed medications (diuretics, spironolactone, ACE inhibitors, angiotensin-II receptor blockers, beta blockers, calcium antagonists, aspirin, and statins) was recorded.

#### Anemia

K-DOQI guidelines (31) indicate a preference for hemoglobin over hematocrit for the determination of anemia. Therefore, plasma hemoglobin was determined using the Siemens ADVIA 120 Hematology system in the hospitals’ central Clinical Chemistry & Hematology Laboratory. World Health Organization (WHO) guidelines indicate that hemoglobin levels of ≤12 g/dL (7.5 mmol/L) for women and <13 g/dL (8.1 mmol/L) for men are indicative of anemia. However, the results of a recent meta-analysis revealed that heterogeneity in reported prevalence across the studies in question primarily resulted from variations in the definition of anemia [5] used. Since there is no clear and standardized definition of anemia in HF patients, we used hemoglobin levels for the main regression analyses. For the analysis on anemia, the WHO diagnostic rules for anemia were used [32].

#### Renal dysfunction

Creatinine levels were determined by the Siemens ADVIA 1650 Clinical chemistry system and were used to evaluate the extent of kidney dysfunction. The MDRD equation was used to calculate the glomerular filtration rate of creatinine (GFR_creat_) [33]. Following the K-DOQI-guidelines, renal dysfunction was defined as a GFR_creat_ of <60 mL/min per 1.73 m^2^ [34].

#### Affective symptoms

The Symptoms of Mixed Anxiety-Depression index (SAD^4^) [35] was used to determine mixed anxiety-depression symptomatology involving two anxiety items (tension, restlessness) and two depression items (feeling blue, hopelessness). Items are answered using a 5-point Likert scale, ranging from 0 (‘not at all’) to 4 (‘very much’). The SAD^4^ showed a high degree of correlation with the STAI anxiety scale (*r* = .69) and the BDI depression scale (*r* = .71) in a sample of myocardial infarction patients [35]. In patients with myocardial infarction a high score on the SAD^4^ (≥3 (upper tertile)) was associated with a substantially increased risk of depressive disorder and/or anxiety disorder [35]. The SAD^4^ has also been used as a predictor of mortality in HF [36], as well as a screening tool for mixed anxiety-depression symptoms in healthy populations [37] and in pregnant women [38]. In the current sample, Cronbach’s alpha for this 4-item scale was .89 and the 1-year test-retest correlation was .75.

#### Type D personality

The 14-item Type D scale (DS14) was used to assess Type D personality [22]. Type D personality is defined as the combination of the negative affectivity and social inhibition personality traits. Individuals with a Type D personality tend to experience negative emotions across time and situations, and have the tendency not to express themselves in social interaction, because of fear of rejection or disapproval by others. The DS14 consists of two 7-item subscales, Negative Affectivity and Social Inhibition that are internally consistent (Cronbach’s α = .88/.86) and independent of health status [22]. Items are answered using a 5-point Likert scale, ranging from 0 (‘false’) to 4 (‘true’). The standardized cut-off score ≥10 on both subscales was used to classify individuals with a Type D personality [22]. Type D personality has shown to be stable over an 18-month period and is not confounded by indicators of disease severity such as LVEF [39], [40] or BNP [41].

### Statistical Analyses

Discrete variables were compared with Chi-square tests and a Fischer’s exact test, when appropriate. Student’s *t*-tests for independent samples were used to compare continuous variables at inclusion. Educational level (primary vs. secondary or higher), marital status (single vs. having a partner), and NYHA class (I/II vs. III) were dichotomized. Hemoglobin levels and creatinine were checked for outliers (M±3SD), which were excluded from further analyses (2 values were omitted for hemoglobin at the 12-month follow-up assessment while 5 values were omitted for creatinine both at inclusion and 12-month follow-up). Skewness was acceptable for both markers, and transformations were not required. At inclusion, blood data were available for 254 patients (due to missing data for hemoglobin (n = 54) and creatinine (n = 26); 16 patients had no values for either). The prospective regression analyses were based on 264 patients, due to missing information on Type D status or affective symptomatology (n = 6) or hemoglobin levels (including outliers) at 12-month follow-up (n = 49).

A Student’s *t*-test was used to assess differences in hemoglobin levels associated with Type D at inclusion. Chi-square tests were used to examine the association between Type D, significant affective symptomatology and anemia at inclusion and follow-up. Logistic regression including a set of clinical variables was used predict changes in anemia status during follow-up.

To investigate Type D personality and affective symptomatology as independent associates of hemoglobin levels at 12-month follow-up, two multivariable regression models were tested (after testing both independent variables in separate unadjusted models). These models included other known contributors to anemia risk as statistical covariates, such as kidney dysfunction (GFR_creat_, diuretics use), NYHA class, RAAS medication (ACE inhibitor or angiotensin receptor blocker), BMI and gender. Finally, the affective symptomatology score was added, to determine whether the personality effect was independent of mood. This final step was repeated for the dichotomized variable, indicating significant affective symptomatology (SAD^4^>3). A Sobel mediation test was used to assess whether covariates that significantly reduced the relation between Type D or affective symptomatology and future hemoglobin were mediators of the relationship. Controls for baseline hemoglobin levels were omitted, since this would overcorrect the regression model and introduce multicollinearity, due to the high degree of correlation with hemoglobin levels at follow-up (dependent variable). Statistical analyses were performed using PASW 19 for Windows (IBM SPSS Inc. Chicago, Illinois, USA). All tests were two-tailed, and α-level of.05 was used to indicate statistical significance.

## Results

### Sample Characteristics

The prevalence of Type D personality was 21% and significant affective symptomatology was present in 34% of the patients. On average, 7.2±4.5 years had passed since the patients were first diagnosed with heart failure. The Type D patients did not differ significantly from non-Type D patients in terms of demographics, biomedical risk factors, disease characteristics, cardiovascular interventions, or prescribed medications at inclusion. Sample characteristics stratified by Type D personality are presented in Table 1.

**Table 1:** Sample characteristics stratified by Type D personality^a^.

	Total(*n* = 313)	Type D(*n* = 64)	non-Type D (*n* = 249)	*p*
*Demographics*				
Male gender	75.3 (235)	71.9 (46)	76.2 (189)	.47
Age (yrs), *mean (SD)*	65.9 (9.9)	67.2 (10.4)	65.5 (9.8)	.25
Living without a partner	25.6 (80)	29.7 (19)	24.6 (61)	.41
*Affective symptoms*				
SAD^4^ score, mean (SD)	2.4 (3.1)	5.4 (3.9)	1.6 (2.3)	**<.0005**
SAD^4^≥3	33.6 (185)	78.1 (50)	22.0 (54)	**<.0005**
*Biomedical risk factors*				
BMI (kg/m^2^) b	27.9 (5.0)	28.0 (5.5)	27.9 (4.9)	.89
Smoking	22.8 (71)	15.6 (10)	24.6 (61)	.13
Hypertension	35.6 (111)	37.5 (24)	35.1 (87)	.72
Hypercholesterolemia	54.8 (171)	57.8 (37)	54.0 (134)	.59
Diabetes	23.7 (74)	26.6 (17)	23.0 (57)	.55
*Disease characteristics*				
Ischemic etiology b	58.1 (180)	53.2 (33)	59.3 (147)	.39
LVEF, mean (SD)	31.7 (6.7)	32.2 (6.3)	31.6 (6.8)	.53
NYHA class III c	31.7 (99)	35.9 (23)	30.6 (76)	.42
Time since diagnosis (yrs), *mean (SD)* b	7.2 (4.5)	7.7 (5.2)	7.0 (4.2)	.23
*Interventions*				
PCI	16.0 (50)	15.6 (10)	16.1 (40)	.92
CABG	26.9 (84)	29.7 (19)	26.2 (65)	.58
Device therapy d	12.5 (39)	12.5 (8)	12.5 (31)	.99
*Prescribed medications*				
Diuretics^e^				
Lisdiuretics only	62.2 (194)	76.6 (49)	58.5 (145)	
Thiazides only	3.2 (10)	1.6 (1)	3.6 (9)	.06
Combined	6.1 (19)	4.7 (3)	6.5 (16)	
Beta blockers	66.5 (206)	68.3 (43)	66.0 (163)	.73
ACE inhibitors	71.8 (224)	75.0 (48)	71.0 (176)	.52
ARB	19.9 (62)	21.9 (14)	19.4 (48)	.65
Digoxin	25.3 (79)	32.8 (21)	23.4 (58)	.12
Calcium antagonists	13.5 (42)	25.0 (16)	10.5 (26)	**.002**
Oral anticoagulants	47.4 (148)	40.6 (26)	49.2 (122)	.22
Aspirin	38.1 (93)	40.6 (26)	37.5 (93)	.65
Statins	53.5 (167)	53.1 (34)	53.6 (133)	.94
Psychotropic medication	13.8 (43)	18.8 (12)	12.5 (31)	.20
*Renal function*				
Renal dysfunction at baseline f	30.4 (95)	31.3 (20)	30.1 (75)	.86
GFR_creat_ at baseline *mean (SD)*	69.8 (20.3)	69.9 (19.8)	69.8 (23.4)	.98
Change in GFR_creat_ during follow-up *mean (SD)*	−1.6 (15.1)	−8.2 (19.7)	−0.1 (13.6)	**.02**

a Results are presented as % (n), unless stated otherwise.

b Due to missing data for 2–23 patients, analyses were conducted using the available data.

c NYHA class III versus NYHA class I-II.

d Either a single, biventricular pacemaker or an implantable cardioverter device.

e 28.1% of patients were not prescribed diuretics. Some patients were prescribed a combination of potassium-sparing diuretics and lisdiuretics (*n* = 12, 3.6%).

f GFR_creat_<60 mL/min per 1.73 m^2.^

ARB = angiotensin-II receptor blockers; BMI = body mass index; CABG = coronary artery bypass grafting; LVEF = left ventricular ejection fraction; NYHA = New York Heart Association functional class; PCI = percutaneous coronary intervention; GFR_creat_ = glomerular filtration rate of creatinine.

In addition, there were no significant differences in the prevalence of cerebrovascular accidents (overall prevalence 9%; *p* = .21), transient ischemic attacks (overall prevalence 9%; *p* = .39), peripheral arterial disease (overall prevalence 16%; *p* = .94), COPD (overall prevalence 14%; *p* = .96), liver disease (overall prevalence 4%; *p* = .93) or gastrointestinal disease (overall prevalence 7%; *p* = .06, with 13% vs. 6% more prevalent in Type Ds).

### Hemoglobin and Anemia Prevalence

**Figure 1:**
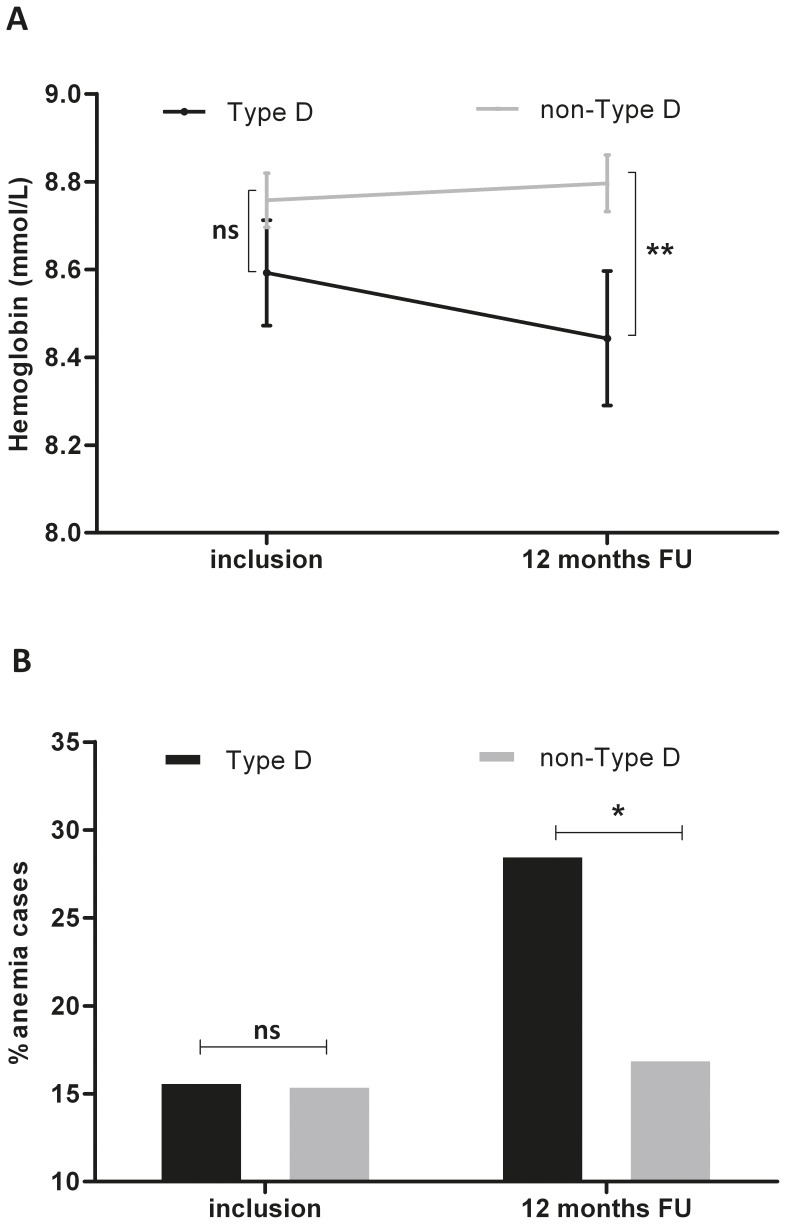
a-b Hemoglobin (1a) and anemia (1b) at inclusion and follow-up by Type D personality. Levels of hemoglobin and prevalence of anemia at inclusion and follow-up by Type D personality. **Figure 1A**: Hemoglobin levels at inclusion and 12 months follow-up (FU) stratified by Type D personality; Grey line = non-Type D patients, black line = Type D patients; the error bars represent 1 standard error of the mean. **Figure 1B**: Percentage of patients with an anemia diagnosis according to WHO guidelines ( = anemia cases), stratified by Type D personality; Grey bars = non-Type D patients, black bar = Type D patients. ** = p<.05 * = p<.10, ns = non-significant.

Hemoglobin levels at inclusion were similar for non-Type D and Type D HF patients (t = 1.2, p = .23) as well as for patients with and without significant affective symptomatology (t = 1.8, p = .08). At 12-month follow-up, hemoglobin levels significantly differed as a function of Type D personality (t = 2.28, p = .02; Figure 1a), but differed only marginally as a function of affective symptomatology (t = 1.9, p = .06). Levels of affective symptomatology were modestly negatively correlated with hemoglobin levels both at baseline (*r* = –.14, p = .02) and 12-month follow-up (*r* = –.11, p = .08). According to WHO criteria, the prevalence of anemia at inclusion was 16% in both non-Type D and Type D patients. However, at 12 months, 17% of non-Type Ds and 29% of Type D patients were classified as anemic (See Figure 1b; χ^2^ = 3.51, p = .06). Regarding clinical predictors of change in anemia status over the one-year period, logistic regression analysis showed that female gender, presence of renal dysfunction at baseline in the absence of anemia, deterioration of renal function, NYHA functional class III, and a longer HF history were predictive of developing anemia (Table 2). Higher baseline hemoglobin levels were protective for becoming anemic.

**Table 2:** Clinical predictors of change in anemia status over 1-year follow-up.

	B	SE	Wald	P value	OR	95% CI
Time since diagnosis	0.2	0.06	6.77	**.009**	**1.17**	1.04–1.32
Hemoglobin at inclusion	−3.1	0.7	20.19	**<.001**	**0.05**	0.01–0.18
BMI	0.1	0.02	0.27	.60	1.01	0.97–1.06
Change in GFR_creat_	0.1	0.03	11.22	**.001**	**1.09**	1.04–1.15
Renal dysfunction at inclusion	1.9	0.7	8.58	**.003**	**6.90**	1.90–25.15
NYHA class III	0.1	0.7	0.02	.89	1.11	0.28–4.38
Female gender	2.9	0.9	10.08	**.001**	**17.9**	3.0–106.4

BMI = body mass index; GFR_creat_ = glomerular filtration rate of creatinine; NYHA class = New York Heart Association functional class.

### Type D Personality, Affective Symptoms and Hemoglobin at 12-month Follow-up

In univariate analysis, Type D personality was significantly associated with future hemoglobin levels (β = –.18, p = .005). Results of the covariate-adjusted analysis showed that Type D personality remained an independent associate of decreased hemoglobin levels at 12-month follow-up (β = –.16, p = .007). In addition, renal dysfunction, gender, and NYHA class were associated with hemoglobin levels at 12-month follow-up (see Table 3 for details of the fully adjusted model, including affective symptomatology). Collinearity statistics showed that tolerance was well within the acceptable range, and that inter-correlation between predictors was low (*r* = –.23 to.17). In total, 25% of variance was accounted for by the variables in the model, which is in keeping with a large total effect size (f^2^ = .36). The change in R^2^ when Type D personality was added to the model was.011, adding.013 to the effect size, an effect equal in proportion to the effect of NYHA class (which added 0.03 to the effect size).

**Table 3:** Independent associates of hemoglobin levels at 12-month follow-up.

	Standardized β	t	*p*
Type D personality	−.15	−2.29	.02
Female gender	−.29	−4.96	<.001
Renal dysfunction at inclusion	−.28	−4.93	<.001
NYHA class	−.12	−1.97	.05
RAAS medication	−.03	−0.45	.66
BMI	.04	0.76	.45
Time since diagnosis	−.12	−2.04	.04
Affective symptomatology	−.01	−0.10	.92

NYHA: New York Heart Association; BMI: body mass index; RAAS: renin-angiotensin-aldosterone system.

In univariate analyses, a higher level of affective symptomatology was prospectively associated with lower hemoglobin levels (β = –.13, p = .04 and β = –.13, p = .045, for continuous and dichotomized affective scores, respectively). However, in adjusted analyses, this association was no longer significant (β = –.09, p = .13 and β = –.09, p = .12, for continuous and dichotomized scores), as gender and renal disease attenuated the link between affective symptomatology and hemoglobin levels. Finally, adding affective symptomatology to the fully adjusted Type D model did not change the effect of Type D personality on future hemoglobin levels (Table 3).

### Type D Personality and Deterioration of Renal Function

Type D was also associated with a larger deterioration in renal function as indicated by a greater change in GFR_creat_ over the follow-up period in Type D patients as compared to non-Type Ds, p = .02 (Table 1). Based on the logistic regression model of clinical predictors of change in anemia status (Table 2), deterioration of renal function was seen as a potential explanatory mechanism, one that could mediate the relation between Type D and future levels of hemoglobin. We employed the Sobel test for mediation analysis, which showed that change in GFR_creat_ was a predictor of lower hemoglobin levels at follow-up (β = –.22, p = .001). It also explained the association between Type D and future lower hemoglobin levels (Type D effect in this model: β = –.05, p = .47).

## Discussion

Type D personality was prevalent in 21% of the current patient sample, and significant affective symptomatology was present in 34% of the patients. Type D personality was associated with lower hemoglobin levels at 12-months follow-up, independent of baseline renal dysfunction, gender, time since diagnosis, medication affecting the renin-angiotensin system, body mass index and NYHA functional class. Affective symptomatology was related to future hemoglobin levels in univariate analyses but not in adjusted analysis. Moreover, adding affective symptomatology to the regression model had no impact on the effect of Type D personality. Importantly, Type D personality was also associated with deterioration of renal function over the year of follow-up, and a Sobel mediation test showed that deterioration of renal function explained the association between Type D and future hemoglobin levels.

The present findings are in line with studies in patients with cancer [18], [20], [21], or COPD [15], and in community-dwelling elderly populations [16], [17], which have shown that increased psychological stress, depressed mood and diminished quality of life were associated with anemia or decreased hemoglobin levels. The current results also build on previous findings in rodents relating psychological stress to plasma iron deficiency and diminished erythropoiesis [13], and on a clinical study which showed that the erythropoietin analogue Darbepoetin-α had a beneficial effect on depressive symptoms in HF patients [19]. Interestingly, the treatment of anemia has been associated with an improvement in quality of life and a depressed mood [21].

Inflammatory activation is one of the mechanisms by which Type D personality might affect hemoglobin levels and renal function. HF patients with Type D personality are characterized by increased levels of pro-inflammatory cytokines [23], [42], as is depression [24], [43]. The pro-inflammatory state is an essential component of anemia or chronic disease [44], as TNF-α and IL-6 inhibit erythropoietin production in the kidney [45] and the proliferation of bone marrow erythroid progenitor cells [46]. Increased levels of pro-inflammatory cytokines have also been associated with poor renal function [47] and a further decline in renal function over time [48]. Future studies are needed to determine whether inflammation could be an explanatory mechanism for the association between psychological distress and lower hemoglobin levels.

Both depression and Type D personality have been associated with deregulated HPA axis activity, resulting in higher daytime cortisol levels [28], [29]. Chronically elevated cortisol may directly suppress erythropoiesis [25], potentially by suppressing the expression of erythropoietin mRNA in the kidneys [26], and could also affect iron metabolism [27], resulting in lower hemoglobin levels [31]. Cortisol could indirectly affect hemoglobin levels by promoting a pro-inflammatory state, as chronically elevated cortisol levels boost the secretion of pro-inflammatory cytokines [49]. Future studies should determine whether cortisol explains the association between Type D personality and hemoglobin.

Behavioral mechanisms that might affect hemoglobin levels include poor self-care and poor medication adherence. Non-compliance with guidelines regarding fluid intake or with diuretic treatment may induce hemodilution, thereby cutting hemoglobin levels. Type D personality has been related to poor medication adherence in patients with acute coronary syndrome [50], but not with poor self-management of health behaviors in heart failure patients [51]. In the current study, Type D was not associated with any changes in the prescription of diuretics or RAAS medication over the follow-up period. However, we have no information on the actual medication taken by these patients.

The present study underscores the clinical relevance of anemia in HF and suggests that psychological distress may be an important factor. Given that anemia in HF is associated with increased mortality, while the correction of anemia might prevent an impaired outcome [52], the close monitoring and targeting of hemoglobin levels might be a promising avenue for efforts to improve clinical outcomes. HF patients with a Type D personality are twice as likely as their non-Type D counterparts to fail to consult a physician in connection with their HF symptoms [51]. Moreover, progressive renal dysfunction largely explained the relation between Type D and a decrease in hemoglobin levels. Therefore, close monitoring of HF and renal symptoms seem to be particularly important in Type D patients. Further research is needed to determine whether targeting hemoglobin in emotionally distressed patients could improve their chances of survival. Some trials (CREATE, CHOIR) have reported that, in the presence of renal failure, increasing the level of hemoglobin may have several deleterious side-effects, such as vasoconstriction and venous thrombosis. These findings could challenge the benefits of anemia correction in HF [53], [54]. It is difficult to extrapolate these results to HF patients as these trials focused primarily on renal failure, and only a subsample of participants was diagnosed with HF [55]. A recent meta-analysis of ESP treatment for anemia in HF concluded that such treatment did not induce these side effects (hypertension, venous thrombosis) or adverse events. On average, however, renal function in these studies was better than in the CREATE and CHOIR trials [55]. As a decline in renal function was a key characteristic of Type D patients with HF, this needs to be carefully investigated in future research. Improving iron deficiency in anemic HF patients (FAIR-HF) enhanced their quality of life while alleviating depressed and anxious moods. However, these benefits were unrelated to the change in anemia [56]. This is in keeping with the current findings for affective symptomatology, which has been shown to be unrelated to anemia, when controlling for disease severity, renal dysfunction and angiotensin-related medication.

A previous study in cancer patients showed that depressed mood and poor quality of life impinged on the effectiveness of erythropoietin-α therapy for anemia. The present study also supports previous recommendations on screening for psychological factors, in order to obtain a fuller understanding of the complex clinical picture of HF [57]. The identification of patients with a Type D personality might benefit the clinical care of HF, as Type D patients are at increased risk of lower hemoglobin levels, and the treatment of anemia may be beneficial in HF.

The results of the current study should be interpreted with appropriate caution, due to several limitations. HF patients are characterized by multi-morbidity and polypharmacy. In the present study, this seems to be slightly more pronounced for some biomedical variables in Type D patients, although not significantly so. It may be that these variables have small, cumulative effects on disease severity and anemia. However, after adjustment for disease severity and renal dysfunction, Type D personality showed an independent association with future hemoglobin. This supports the hypothesis that the effect of psychological stress is of significant added value. No information was available on the dosage of prescribed medications, or on iron blood levels or iron intake. Further, affective symptomatology was assessed by a 4-item screening instrument for mixed anxiety-depression symptoms and not by more standard scales or psychiatric interviews. Since the SAD^4^ asks to report affective symptomatology over the past week, it provides no information on the duration of the reported symptoms. This may imply that the results do not necessarily generalize to depression or anxiety diagnosis. Although the current study design was longitudinal, the lack of a control group means that no final conclusions can be drawn regarding causality. Baseline hemoglobin was not included, due to the potential multicollinearity with follow-up levels of hemoglobin. Finally, no information was available on the activity of the sympathetic nervous system, or the HPA axis, precluding any final conclusions on the pathophysiological mechanisms of effect.

Future research should examine the stress-related mechanisms through which hemoglobin levels are affected in HF patients with Type D personality. Does altered HPA axis function or sympathetic activity explain differences in hemoglobin levels between Type D vs. non-Type D individuals? A prospective, observational cohort study in patients with HF in which basal daytime levels of cortisol, norepinephrine and hemoglobin are collected at multiple measurement occasions would help to resolve the current paucity of knowledge on pathophysiological mechanisms. In addition, further research is needed to determine whether treatment with erythropoiesis-stimulating proteins could improve hemoglobin levels in HF patients with a Type D personality. This could initially be explored in an experimental setting in which erythropoiesis-stimulating proteins are given to Type D and non-Type D anemic individuals without heart disease, before extending the study towards anemic cardiac patients.

In conclusion, Type D personality was associated with lower hemoglobin levels at 12-month follow-up, independent of renal dysfunction, gender, NYHA class, BMI, RAAS medication and affective symptomatology. Type D personality was also associated with deterioration of renal function over the follow-up period, which could explain the decrease in hemoglobin levels observed in Type D patients. Identifying psychological risk factors and their mechanisms of action might enhance our understanding of the complex disease processes involved in HF.
